# Assessing elements of a family approach to reduce adolescent drinking frequency: parent–adolescent relationship, knowledge management and keeping secrets

**DOI:** 10.1111/add.13258

**Published:** 2016-02-10

**Authors:** Mark McCann, Oliver Perra, Aisling McLaughlin, Claire McCartan, Kathryn Higgins

**Affiliations:** ^1^ MRC/CSO Social and Public Health Sciences UnitUniversity of GlasgowGlasgowUK; ^2^ School of Nursing and MidwiferyQueen's University BelfastBelfastUK; ^3^ Institute of Child Care ResearchQueen's University BelfastBelfastUK

**Keywords:** Adolescence, alcohol, child, parental monitoring, parent–child relationship, secrecy

## Abstract

**Aims:**

To estimate (1) the associations between parent–adolescent relationship, parental knowledge and subsequent adolescent drinking frequency and (2) the influence of alcohol use on parental knowledge.

**Design:**

Path analysis of school based cohort study with annual surveys.

**Setting:**

Post‐primary schools from urban and intermediate/rural areas in Northern Ireland.

**Participants:**

A total of 4937 post‐primary school students aged approximately 11 years in 2000 followed until approximately age 16 years in 2005.

**Measurements:**

Pupil‐reported measures of: frequency of alcohol use; parent–child relationship quality; subdimensions of parental monitoring: parental control, parental solicitation, child disclosure and child secrecy.

**Findings:**

Higher levels of parental control [ordinal logistic odds ratio (OR) = 0.86, 95% confidence interval (CI) = 0.78, 0.95] and lower levels of child secrecy (OR = 0.83, 95% CI = 0.75, 0.92) were associated subsequently with less frequent alcohol use. Parental solicitation and parent–child relationship quality were not associated with drinking frequency. Weekly alcohol drinking was associated with higher subsequent secrecy (beta −0.42, 95% CI = –0.53, −0.32) and lower parental control (beta −0.15, 95% CI = –0.26, −0.04). Secrecy was more strongly predictive of alcohol use at younger compared with older ages (*P* = 0.02), and alcohol use was associated less strongly with parental control among families with poorer relationships (*P* = 0.04).

**Conclusions:**

Adolescent alcohol use appears to increase as parental control decreases and child secrecy increases. Greater parental control is associated with less frequent adolescent drinking subsequently, while parent–child attachment and parental solicitation have little influence on alcohol use.

## Introduction

More than 70% of European students have drunk alcohol at least once 1. While alcohol has social functions, excess and early drinking may confer a risk of physical, emotional and social harm. While there may be no causal link between age of onset and later chronic alcohol problems 2, 3, early use is predictive of future drinking patterns 4, which can contribute to long‐term health outcomes 5, 6, 7, delinquency 8, 9, 10, mental health problems and risky sexual behaviour 11, 12 and poor academic performance 8, 13, 14.

Providing emotional support for children is one element of the parental role 15; another key element is parenting behaviours to minimize the risk of physical or social harm 16. Parental monitoring—or parent's ongoing knowledge of their child's activities, whereabouts, etc.—has been proposed as the primary family‐level determinant of alcohol outcomes: low parental monitoring has been associated with adolescent drinking outcomes 17, 18, 19; one study showed that the importance of other family factors is negligible after accounting for parental knowledge 20. Children choose whether or not to disclose information about their activities and to keep secrets from their parents 21. Frijns *et al.* reported that secrecy from parents was an important risk factor for adolescent psychosocial wellbeing and behavioural adjustment 22; the child disclosure–adjustment link may actually reflect a secrecy–maladjustment link 23. Adolescent agency to influence their environment (e.g. via selective information sharing with parents) may be particularly important in households where family relationships are already strained. Not only may parents change monitoring behaviour, but children may also choose different communication strategies depending on the quality of the relationship 24. Few studies have been in a position to assess how alcohol relates to adolescent agency, or the extent to which young people influence their family environment actively.

While it is conceptually plausible that parent–child relationship could influence drinking (e.g. in response to stress 25 or hindered development of coping skills 15), there is little evidence for a prospective association between parent–child relationship and drinking 26, 27. Relationship quality could influence monitoring patterns, and potentially the association between monitoring and child outcomes 28, so parental monitoring may be associated more strongly with alcohol use among children with poor family relationships and thus greater propensity to drink. By comparison, for adolescents with a more positive home environment, monitoring may not play as important a role.

Using longitudinal data across the period from early to mid‐adolescence, this study will assess the relationships between parent–child attachment, parent and child behaviours influencing monitoring and adolescent alcohol use. It will also assess how associations differ according to home environment. Given the known gender differences in alcohol use 29 and in parent–child relationships 30, 31, we will also assess differences by sex of the child. Our research questions are:

1. Does the quality of parent–child relationship and levels of parental monitoring affect alcohol use frequency?
2. Does alcohol use frequency influence subsequent levels of parental monitoring?
3. Does the monitoring and alcohol dynamic vary comparing high versus low attached families?
4. Do the associations change from early to late adolescence?

## Methods

This paper used data from the Belfast Youth Development Study, a longitudinal study of adolescent substance use. Between 2000 and 2011, children attending approximately 40 schools, colleges and special educational programmes across Northern Ireland were given questionnaires on a range of issues, including substance use and family life. Pupils were in their first year of secondary school (approximately age 11) at the start of the study (academic year 2000/2001), and were surveyed annually until 2006/2007 (approximately age 17) whether or not in education, again approximately 10 years after the first sweep (2011). This report is based on data from the first 5 years of the study. There were 6156 pupils ever registered at participating schools during the study period. After excluding those who did not provide data at any time‐point, withdrew from the study or those whose parents did not consent for them to participate, data were available for 5209 (85%) individuals. As this study focused on parent–child relationships, 195 children who stated that they did not live with their parents were removed from the analysis, leaving 5014 individuals. There were 77 individuals who did not complete the Inventory of Peer and Parental Attachment (IPPA) scale at any time‐point. They were also dropped from the analysis, leaving 4937 individuals in the final analysis.

### Study variables

Mental health was measured using the Strengths and Difficulties Questionnaire (SDQ) 32, a mental health screener for children and adolescents. The SDQ was completed in years 1 and 4. For family affluence, respondents were asked about the number of cars at their household, number of family holidays, parental employment, if they have their own bedroom, the type of house they lived in and eligibility for free school meals. Affluence measures were based on principal components analysis of these items 33. Regarding living arrangements, respondents were asked with whom they lived, and responses were grouped into: both biological parents; step/foster‐family (one biological and one step/foster‐parent); and single‐parent. Respondents in other households (predominantly siblings or grandparents) were excluded. Living arrangements in year 5 of the study were used in the analysis. If this information was missing in year 5, the previous year's data were used.

#### Analytical variables

Each year, participants were asked about how frequently they drank alcohol. Responses for each year were coded as: does not drink (0); rarely drinks (1); drinks monthly (2); and drinks weekly or more frequently 3. Stattin & Kerr's (2000) measures of parental monitoring were asked in each year. Rather than looking at the overall monitoring measure, we focused on the mechanisms by which parents obtain information. The parental solicitation component enquired about how much parents ask their children about what they do; parental control relates to needing parents' permission or parental demands for information. While developed originally as a single scale, the child disclosure component has been shown to comprise two factors 23. The first centres on information offered to parents, referred to henceforth as ‘disclosure', and the second centres on withholding information from parents, referred to as ‘secrecy'. Higher scores on these scales indicated more ‘positive' ratings, i.e. higher levels of parental control and solicitation, higher child disclosure or lower levels of secrecy (i.e. keeping fewer secrets). The inventory of peer and parental attachment 34 was also included in the analysis: this scale includes questions such as ‘my parents respect my feelings' and ‘I trust my parents'. The IPPA comprised 12 items on a three‐point scale in year 1, while in years 3 and 4 it comprised 28 items on a five‐point scale. Higher scores indicate better parent–child relationship. Respondents were split into high‐ and low‐attached groups based on IPPA.

#### Statistical analysis

**Figure 1:**
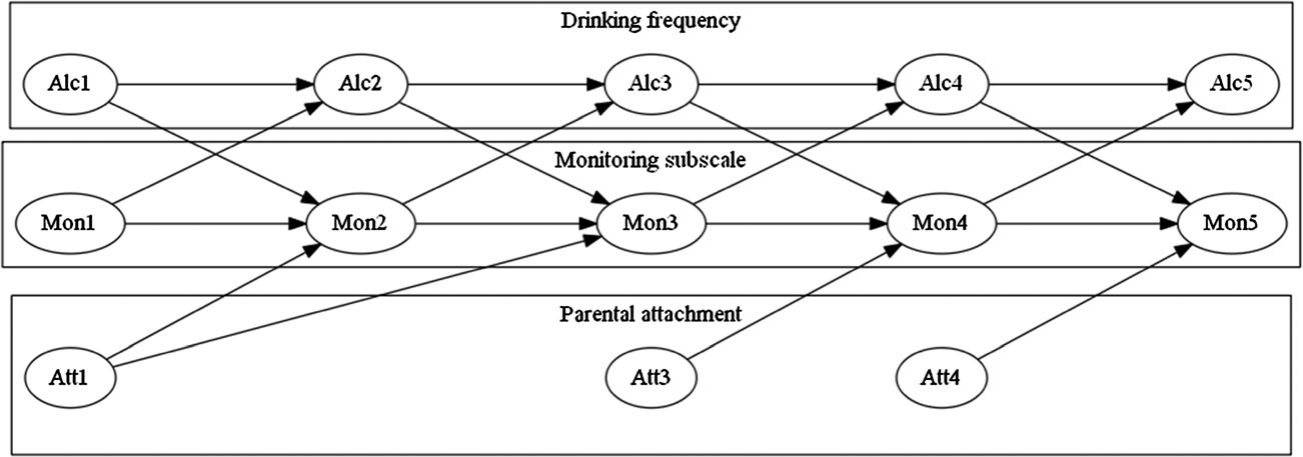
Representation of the path models resting the association between monitoring and alcohol use. Att = attachment, Alc = alcohol drinking frequency, Mon = parental monitoring

Cross‐lagged path analysis assessed the association between alcohol use and monitoring dimensions over time. Monitoring and alcohol use were regressed on variables at a previous time‐point. For alcohol use and monitoring this was always a 1‐year lag; for SDQ and IPPA, the lag was to the last year they were asked (years 1, 3 or 4). The models contained the following components: (a) cross‐sectional correlations between year 1 variables; (b) stability paths, the association between the same variable from one time‐point to the next; and (c) cross‐lagged paths, showing the association between one variable and subsequent levels of the other variables (see Fig. 1) These cross‐lagged paths suggest to what extent alcohol use or monitoring is associated with subsequent change in the other construct, over and above that expected given their prior levels of drinking or monitoring. These models were fitted separately for each monitoring subcomponent; after this, the full model retaining all influential components was fitted. For this full model, subgroup analysis then estimated if stability and cross‐lagged paths differed between (d) males and females and (e) high‐attachment and low‐attachment families. Finally, (f) the full cohort model was used to assess if the cross‐lagged and stability paths differed across each study year. All reported coefficients were conditioned on gender, affluence, SDQ, IPPA and living arrangements, and models were estimated using robust standard errors accounting for clustering at the school level; information was not collected on classes within schools. The Appendix contains further details on variables and the analyses of between‐group differences. Latent profile analyses were conducted in MPlus 7, while the main analyses were perfumed using Stata version 13.1. The Sem and gsem routines were used in conjunction with mi (multiple imputation), while multiple group and time trend tests were conducted using mi testtransform; these results appear in the Supporting information Appendix. Results below are based on Stata's regress and ologit routines.

## Results

A total of 4937 were included into the study; 2348 (48%) were male and 2589 (52%) were female. Approximately 8–9% of the cohort showed some signs of mental health problems at both time‐points that they were assessed. Approximately 71% (3545) of the cohort lived with both biological parents, 19% (942) lived with one parent only and approximately 9% (450) lived in a different household, e.g. parent and parent's partner, with foster‐parents, etc. The mean [standard deviation (SD)] IPPA scores were 29.9 (3.8), 103.4 (21.8) and 102.9 (22.6) in years 1, 3 and 4, respectively.

A greater proportion of boys reported frequent drinking in year 1 (8% of males reported drinking weekly or more frequently compared to 3% of females), but in the following years there was little gender difference. Those living with step‐parent or single‐parent families drank more frequently from an earlier age than those with two biological parents, as did those with poorer mental health and less secure parental attachment.

### Path models

Fit statistics that are appropriate to clustered data indicated reasonable model fit 35 (see Supporting information Appendix, Table A5). Tables 1, 2, 3, 4 show the coefficients for the bidirectional associations between alcohol use and parental control, child disclosure, child secrecy and parental solicitation, respectively. Parental disclosure showed little association with alcohol use and was dropped from further models. Table 5 shows the mutually adjusted model testing cross‐lagged paths between alcohol, parental control, child disclosure and secrecy. Tables showing tests for time trends, gender differences and parental attachment group differences are reported in the Supporting information Appendix.

**Table 1:** Cross‐lagged coefficients for the association between alcohol use frequency and parental control.

*Path*	*Coefficient (95% CI)*	*Path*	*Coefficient (95% CI)*	*Path*	*Coefficient (95% CI)*	*Path*	*Coefficient (95% CI)*
**Year 2 Alcohol**		**Year 3 Alcohol**		**Year 4 Alcohol**		**Year 5 Alcohol**	
Year 1 Control	0.77 (0.72, 0.83)	Year 2 Control	0.76 (0.71, 0.81)	Year 3 Control	0.77 (0.72, 0.83)	Year 4 Control	0.84 (0.77, 0.92)
Year Attachment	0.96 (0.89, 1.04)	Year 1 Attachment	0.91 (0.85, 0.97)	Year 3 Attachment	0.965 (0.90, 1.04)	Year 4 Attachment	0.98 (0.89, 1.07)
Year 2 Control		Year 3 Control		Year 4 Control		Year 5 Control	
Year 1 Alcohol		Year 2 Alcohol		Year 3 Alcohol		Year 4 Alcohol	
None	Reference	None	Reference	None	Reference	None	Reference
Rarely	−0.11 (−0.18, −0.04)	Rarely	−0.09 (−0.15, −0.03)	Rarely	−0.08 (−0.15, −0.01)	Rarely	−0.04 (−0.13, 0.06)
Monthly	−0.111 (−0.27, 0.04)	Monthly	−0.17 (−0.26, −0.08)	Monthly	−0.11 (−0.20, −0.03)	Monthly	−0.09 (−0.19, 0.01)
Weekly or more	−0.197 (−0.36, −0.03)	Weekly or more	−0.262 (−0.35, −0.18)	Weekly or more	−0.18 (−0.26, −0.09)	Weekly or more	−0.19 (−0.30, −0.09)
Year 1 Attachment	0.07 (0.04, 0.11)	Year 1 Attachment	0.10 (0.06, 0.13)	Year 3 Attachment	0.05 (0.01, 0.08)	Year 4 Attachment	0.01 (−0.03, 0.04)

Coefficients for alcohol represent ordinal logistic regression odds ratios; coefficients for control represent linear regression standardized beta coefficients; coefficients adjusted for sex, living arrangements, affluence and mental health. CI = confidence interval. Coefficients in bold denote P < 0.05. Bold text denotes outcome variable.

**Table 2:** Cross‐lagged coefficients for the association between alcohol use frequency and child disclosure.

*Path*	*Coefficient (95% CI)*	*Path*	*Coefficient (95% CI)*	*Path*	*Coefficient (95% CI)*	*Path*	*Coefficient (95% CI)*
**Year 2 Alcohol**		**Year 3 Alcohol**		**Year 4 Alcohol**		**Year 5 Alcohol**	
Year 1Disclosure	0.77 (0.71, 0.83)	Year 2 Disclosure	0.80 (0.74, 0.85)	Year 3 Disclosure	0.77 (0.71, 0.83)	Year 4 Disclosure	0.87 (0.80, 0.96)
Year 1 Attachment	0.99 (0.92, 1.07)	Year 1 Attachment	0.92 (0.86, 0.99)	Year 3 Attachment	1.04 (0.96, 1.12)	Year 4 Attachment	1.01 (0.91, 1.12)
Year 2 Disclosure		Year 3 Disclosure		Year 4 Disclosure		Year 5 Disclosure	
Year 1 Alcohol		Year 2 Alcohol		Year 3 Alcohol		Year 4 Alcohol	
None	Reference	None	Reference	None	Reference	None	Reference
Rarely	−0.15 (−0.22, −0.08)	Rarely	−0.10 (−0.16, −0.04)	Rarely	−0.13 (−0.22, −0.04)	Rarely	−0.12 (−0.21, −0.03)
Monthly	−0.29 (−0.45, −0.14)	Monthly	−0.17 (−0.25, −0.08)	Monthly	−0.24 (−0.34, −0.14)	Monthly	−0.23 (−0.33, −0.13)
Weekly or more	−0.22 (−0.39, −0.06)	Weekly or more	−0.17 (−0.26, −0.08)	Weekly or more	−0.23 (−0.33, −0.14)	Weekly or more	−0.23 (−0.33, −0.13)
Year 1 Attachment	0.13 (0.09, 0.17)	Year 1 Attachment	0.13 (0.09, 0.16)	Year 3 Attachment	0.14 (0.11, 0.17)	Year 4 Attachment	0.19 (0.15, 0.23)

Coefficients for alcohol represent ordinal logistic regression odds ratios; coefficients for disclosure represent linear regression standardized beta coefficients; coefficients adjusted for sex, living arrangements, affluence and mental health. CI = confidence interval. Coefficients in bold denote P < 0.05. Bold text denotes outcome variable.

**Table 3:** Cross‐lagged coefficients for the association between alcohol use frequency and child secrecy.

*Path*	*Coefficient (95% CI)*	*Path*	*Coefficient (95% CI)*	*Path*	*Coefficient (95% CI)*	*Path*	*Coefficient (95% CI)*
**Year 2 Alcohol**		**Year 3 Alcohol**		**Year 4 Alcohol**		**Year 5 Alcohol**	
Year 1 Secrecy	0.71 (0.65, 0.76)	Year 2 Secrecy	0.77 (0.72, 0.83)	Year 3 Secrecy	0.73 (0.67, 0.79)	Year 4 Secrecy	0.81 (0.73, 0.90)
Year 1 Attachment	1.00 (0.92, 1.08)	Year 1 Attachment	0.91 (0.84, 0.97)	Year 3 Attachment	1.01 (0.93, 1.09)	Year 4 Attachment	1.02 (0.92, 1.12)
Year 2 Secrecy		Year 3 Secrecy		Year 4 Secrecy		Year 5 Secrecy	
Year 1 Alcohol		Year 2 Alcohol		Year 3 Alcohol		Year 4 Alcohol	
None	Reference	None	Reference	None	Reference	None	Reference
Rarely	−0.21 (−0.29, −0.14)	Rarely	−0.21 (−0.28, −0.15)	Rarely	−0.19 (−0.26, −0.11)	Rarely	−0.33 (−0.42, −0.24)
Monthly	−0.32 (−0.48, −0.15)	Monthly	−0.33 (−0.43, −0.24)	Monthly	−0.31 (−0.40, −0.22)	Monthly	−0.47 (−0.57, −0.36)
Weekly or more	−0.41 (−0.57, −0.25)	Weekly or more	−0.40 (−0.50, −0.30)	Weekly or more	−0.39 (−0.48, −0.30)	Weekly or more	−0.54 (−0.65, −0.44)
Year 1 Attachment	0.09 (0.05, 0.13)	Year 1 Attachment	0.10 (0.06, 0.14)	Year 3 Attachment	0.14 (0.10, 0.17)	Year 4 Attachment	0.17 (0.13, 0.21)

Coefficients for alcohol represent ordinal logistic regression odds ratios; coefficients for secrecy represent linear regression standardized beta coefficients. Higher secrecy scores indicate lower levels of secret‐keeping. Coefficients for disclosure represent linear regression standardized beta coefficients. Coefficients adjusted for sex, living arrangements, affluence and mental health. CI = confidence interval. Coefficients in bold denote P < 0.05. Bold text denotes outcome variable.

**Table 4:** Cross‐lagged coefficients for the association between alcohol use frequency and parental solicitation.

*Path*	*Coefficient (95% CI)*	*Path*	*Coefficient (95% CI)*	*Path*	*Coefficient (95% CI)*	*Path*	*Coefficient (95% CI)*
**Year 2 Alcohol**		**Year 3 Alcohol**		**Year 4 Alcohol**		**Year 5 Alcohol**	
Year 1 Solicitation	0.99 (0.92, 1.07)	Year 2 Solicitation	0.92 (0.86, 0.99)	Year 3 Solicitation	1.07 (0.99, 1.15)	Year 4 Solicitation	1.01 (0.93, 1.11)
Year 1 Attachment	0.91 (0.84, 0.98)	Year 1 Attachment	0.89 (0.83, 0.95)	Year 3 Attachment	0.89 (0.82, 0.96)	Year 4 Attachment	0.94 (0.85, 1.04)
Year 2 Solicitation		Year 3 Solicitation		Year 4 Solicitation		Year 5 Solicitation	
Year 1 Alcohol		Year 2 Alcohol		Year 3 Alcohol		Year 4 Alcohol	
None	Reference	None	Reference	None	Reference	None	Reference
Rarely	−0.09 (−0.16, −0.02)	Rarely	0.01 (−0.06, 0.07)	Rarely	0.04 (−0.03, 0.11)	Rarely	0.05 (−0.05, 0.14)
Monthly	−0.20 (−0.36, −0.03)	Monthly	−0.00 (−0.10, 0.09)	Monthly	−0.00 (−0.10, 0.10)	Monthly	0.03 (−0.07, 0.13)
Weekly or more	−0.19 (−0.36, −0.02)	Weekly or more	−0.03 (−0.13, 0.06)	Weekly or more	0.01 (−0.08, 0.09)	Weekly or more	0.08 (−0.03, 0.18)
Year 1 Attachment	0.13 (0.09, 0.17)	Year 1 Attachment	0.13 (0.09, 0.17)	Year 3 Attachment	0.11 (0.08, 0.15)	Year 4 Attachment	0.07 (0.03, 0.10)

Coefficients for alcohol represent ordinal logistic regression odds ratios; coefficients for solicitation represent linear regression standardized beta coefficients; coefficients adjusted for sex, living arrangements, affluence and mental health; coefficients for disclosure represent linear regression standardized beta coefficients. CI = confidence interval. Coefficients in bold denote P < 0.05. Bold text denotes outcome variable.

**Table 5:** Cross‐lagged coefficients for the association between alcohol use frequency, parental control, child disclosure and child secrecy.

*Path*	*Coefficient (95% CI)*	*Path*	*Coefficient (95% CI)*	*Path*	*Coefficient (95% CI)*	*Path*	*Coefficient (95% CI)*
**Year 2 Alcohol**		**Year 3 Alcohol**		**Year 4 Alcohol**		**Year 5 Alcohol**	
Year 1 Control	0.83 (0.77, 0.90)	Year 2 Control	0.81 (0.75, 0.87)	Year 3 Control	0.83 (0.76, 0.90)	Year 4 Control	0.86 (0.78, 0.95)
Year 1 Disclosure	0.89 (0.82, 0.96)	Year 2 Disclosure	0.92 (0.85, 0.99)	Year 3 Disclosure	0.88 (0.80, 0.96)	Year 4 Disclosure	0.96 (0.86, 1.07)
Year 1 Secrecy	0.76 (0.70, 0.82)	Year 2 Secrecy	0.82 (0.76, 0.89)	Year 3 Secrecy	0.76 (0.70, 0.83)	Year 4 Secrecy	0.83 (0.75, 0.92)
Year 1 Attachment	1.06 (0.98, 1.15)	Year 1 Attachment	0.94 (0.88, 1.02)	Year 3 Attachment	1.10 (1.02, 1.19)	Year 4 Attachment	1.06 (0.95, 1.18)
**Year 2 Control**		**Year 3 Control**		**Year 4 Control**		**Year 5 Control**	
Year 1 Alcohol		Year 2 Alcohol		Year 3 Alcohol		Year 4 Alcohol	
None	Reference	None	Reference	None	Reference	None	Reference
Rarely	−0.08 (−0.15, −0.01)	Rarely	−0.06 (−0.12, 0.01)	Rarely	−0.05 (−0.12, 0.02)	Rarely	−0.02 (−0.12, 0.07)
Monthly	−0.04 (−0.19, 0.12)	Monthly	−0.11 (−0.20, −0.02)	Monthly	−0.07 (−0.16, 0.03)	Monthly	−0.06 (−0.17, 0.04)
Weekly or more	−0.11 (−0.28, 0.06)	Weekly or more	−0.17 (−0.27, −0.08)	Weekly or more	−0.11 (−0.20, −0.02)	Weekly or more	−0.15 (−0.26, −0.04)
Year 1 Disclosure	0.06 (0.02, 0.10)	Year 2 Disclosure	0.06 (0.03, 0.10)	Year 3 Disclosure	0.08 (0.05, 0.13)	Year 4 Disclosure	0.07 (0.02, 0.11)
Year 1 Secrecy	0.06 (0.02, 0.10)	Year 2 Secrecy	0.05 (0.02, 0.09)	Year 3 Secrecy	0.05 (0.01, 0.08)	Year 4 Secrecy	0.03 (−0.01, 0.06)
Year 1 Attachment	0.04 (0.01, 0.08)	Year 1 Attachment	0.08 (0.04, 0.11)	Year 3 Attachment	−0.00 (−0.04, 0.03)	Year 4 Attachment	−0.03 (−0.07, 0.01)
**Year 2 Disclosure**		**Year 3 Disclosure**		**Year 4 Disclosure**		**Year 5 Disclosure**	
Year 1 Alcohol		Year 2 Alcohol		Year 3 Alcohol		Year 4 Alcohol	
None	Reference	None	Reference	None	Reference	None	Reference
Rarely	−0.10 (−0.17, −0.03)	Rarely	−0.06 (−0.12, 0.01)	Rarely	−0.04 (−0.10, 0.03)	Rarely	−0.09 (−0.17, 0.00)
Monthly	−0.16 (−0.32, −0.00)	Monthly	−0.10 (−0.19, −0.01)	Monthly	−0.09 (−0.18, −0.00)	Monthly	−0.15 (−0.25, −0.06)
Weekly or more	−0.11 (−0.27, 0.06)	Weekly or more	−0.06 (−0.15, 0.04)	Weekly or more	−0.07 (−0.16, 0.02)	Weekly or more	−0.10 (−0.20, 0.00)
Year 1 Control	0.11 (0.07, 0.15)	Year 2 Control	0.12 (0.08, 0.15)	Year 3 Control	0.10 (0.06, 0.13)	Year 4 Control	0.02 (−0.02, 0.05)
Year 1Secrecy	0.11 (0.07, 0.15)	Year 2 Secrecy	0.09 (0.05, 0.12)	Year 3 Secrecy	0.08 (0.05, 0.11)	Year 4 Secrecy	0.16 (0.12, 0.19)
Year 1 Attachment	0.10 (0.06, 0.14)	Year 1 Attachment	0.11 (0.08, 0.15)	Year 3 Attachment	0.10 (0.07, 0.14)	Year 4 Attachment	0.15 (0.11, 0.19)
**Year 2 Secrecy**		**Year 3 Secrecy**		**Year 4 Secrecy**		**Year 5 Secrecy**	
Year 1 Alcohol		Year 2 Alcohol		Year 3 Alcohol		Year 4 Alcohol	
None	Reference	None	Reference	None	Reference	None	Reference
Rarely	−0.19 (−0.27, −0.12)	Rarely	−0.17 (−0.24, −0.11)	Rarely	−0.17 (−0.24, −0.09)	Rarely	−0.28 (−0.37, −0.19)
Monthly	−0.25 (−0.42, −0.08)	Monthly	−0.27 (−0.37, −0.17)	Monthly	−0.27 (−0.37, −0.18)	Monthly	−0.38 (−0.48, −0.28)
Weekly or more	−0.35 (−0.51, −0.18)	Weekly or more	−0.30 (−0.41, −0.20)	Weekly or more	−0.33 (−0.43, −0.24)	Weekly or more	−0.42 (−0.53, −0.32)
Year 1 Control	0.06 (0.02, 0.10)	Year 2 Control	0.04 (0.00, 0.07)	Year 3 Control	0.02 (−0.01, 0.06)	Year 4 Control	0.06 (0.02, 0.09)
Year 1 Disclosure	0.07 (0.03, 0.11)	Year 2 Disclosure	0.16 (0.12, 0.20)	Year 3 Disclosure	0.11 (0.07, 0.15)	Year 4 Disclosure	0.24 (0.19, 0.28)
Year 1 Attachment	0.07 (0.03, 0.11)	Year 1 Attachment	0.06 (0.02, 0.10)	Year 3 Attachment	0.09 (0.05, 0.13)	Year 4 Attachment	0.07 (0.03, 0.11)

Coefficients for alcohol represent ordinal logistic regression odds ratios; coefficients for control, disclosure and secrecy represent linear regression standardized beta coefficients; coefficients for disclosure represent linear regression standardized beta coefficients; coefficients adjusted for sex, living arrangements, affluence and mental health. CI = confidence interval. Coefficients in bold denote P < 0.05. Bold text denotes outcome variable.

### Influences on frequency of alcohol use

Apart from the parental solicitation subscale, higher levels of parental monitoring were associated with less frequent use of alcohol. When observing a 1 SD increase in parental monitoring in year 4, there was a lower odds of frequent drinking in year 5; this was observed for parental control, beta 0.84 [95% confidence interval (CI) = 0.77, 0.92], child disclosure, beta 0.87 (95% CI = 0.80, 0.96) and child secrecy, beta 0.81 (95% CI = 0.73, 0.90), but there was no change in drinking frequency associated with levels of parental solicitation (beta 1.01, 95% CI = 0.93, 1.11). There was no evidence of variation over time for the influence of parental control (*P* = 0.29) and child disclosure (*P* = 0.53) on alcohol use. The association between child secrecy and alcohol use did appear to vary over time (*P* = 0.02), with less of an association with the secrecy scale in year 1 (*P* = 0.03) than the scale in year 2 (*P* = 0.47). IPPA showed little association with drinking frequency [odds ratio (OR) = 0.94, 95% CI = 0.85, 1.04) and the influence of monitoring did not vary comparing high and low IPPA groups.

### Influences on parental control

More frequent adolescent alcohol use was associated with lower parental control. Respondents drinking weekly or more frequently showed an approximately 0.20 SD reduction in parental control in the subsequent year. This association did not appear to vary over time (*P* = 0.18). Respondents with higher disclosure scores reported slightly higher subsequent parental control, and this did not appear to vary over time (*P* = 0.96); a 1‐unit increase in year 4 disclosure was associated with a 7% increase in parental control (beta 0.07, 95% CI = 0.02, 0.11). Lower levels of child secrecy were associated with higher parental control in the earlier years of the study, but by age 14 the association attenuated to suggest no effect. There was some evidence for a time trend (*P* = 0.09). Parental attachment did not appear to influence parental control (year 4 IPPA standardized beta 0.028, 95% CI = –0.07, 0.01).

### Influences on child disclosure

Alcohol use was associated with lower levels of child disclosure. Compared to non‐drinkers, year 5 disclosure scores were lower for infrequent (beta −0.09, 95% CI = −0.17, 0.00), monthly (beta −0.15, 95% CI = –0.25, −0.06) and weekly (beta −0.10, 95% CI = –0.20, 0.00) drinkers. There was little association of child disclosure with parental control (beta 0.02, 95% CI = 0.02, 0.05), but more of an association with child secrecy (beta 0.16, 95% CI = 0.12, 0.19) and parental attachment (beta 0.15, 95% CI = 0.11, 0.19). The association with alcohol (*P* = 0.17) did not vary over time, while the control–disclosure association did (*P* < 0.001), with stronger evidence of a change in association in the later years of the study (*P* = 0.09), possibly explained by there being little association in year 5. The secrecy–disclosure association also varied over time (*P* < 0.001), again suggesting greater change at the later years.

### Influences on child secrecy

The strongest association found across any of the models was the association between alcohol use and subsequent levels of child secrecy. Compared to non‐drinkers in year 4, the rates of secrecy were higher in year 5: for infrequent drinkers by beta 0.28 (95% CI = 0.37, 0.19), monthly drinkers, beta 0.38 (95% CI = 0.48, 0.28) and weekly drinkers, beta 0.42 (95% CI = 0.53, 0.32). Alcohol use was, in fact, a stronger predictor of subsequent secrecy‐keeping than previous levels of secrecy (beta 0.27, 95% CI = 0.23, 0.31). Higher parental control (beta 0.06, 95% CI = 0.02, 0.09), parent–child attachment (beta 0.07, 95% CI = 0.03, 0.11) and child disclosure (beta 0.24, 95% CI = 0.19, 0.28) were also associated with lower levels of secrecy. The association between disclosure and secrecy appeared to vary over time (*P* < 0.001), with evidence for a change in association between each time‐point. There was no evidence of time trends for alcohol (*P* = 0.33), control (*P* = 0.14) or attachment (*P* = 0.29).

### Variation by gender

Based on tests that the difference in the coefficients between the male and female models was equal to zero, the strongest suggestion of a gender difference was found for the alcohol–parental control association (*P* = 0.08), indicating that alcohol use was associated with a greater reduction in parental control among boys than girls.

### Variation by high versus low parental attachment

There was no evidence of variation between high and low IPPA groups, except for differences in how alcohol use relates to subsequent parental control (*P* = 0.04) and child disclosure (*P* = 0.03). The difference in parental control appeared greatest at older ages (year 5 *P* = 0.01), but at the youngest ages for child disclosure (*P* = 0.03). Alcohol use is followed by a greater reduction in parental control, but a lesser reduction in disclosure among the securely compared to the less securely attached group.

## Discussion

The study demonstrated the association between parental monitoring and adolescent alcohol use. The findings correspond with previous research 17, 36, and additionally highlight the specific roles of parental control, children's withholding information and a reverse causal influence of drinking to reduce levels of monitoring. Parental monitoring and child drinking appear to influence each other. This suggests that interventions targeting changes in parenting practice and child drinking as dynamic processes could have benefits over and above preventing or delaying alcohol use initiation. There was little evidence of differences in these associations by gender or parent–child relationship, and the influence of monitoring on alcohol use was consistent over time.

Alcohol use seemed to have a stronger influence on parental control than on child disclosure, despite the fact that adolescents directly influence their own behaviour, but not that of their parents. There are a number of explanations: (1) children who drink more often may feel less controlled by their parents; (2) age may confound the control–alcohol use relationship; (3) drinking adolescents may have greater incentive to negotiate autonomy and independence, to facilitate socializing in drinking environments; and (4) parents are aware of their children's alcohol use and reduce control as a result. Point 1 does not account for the fact that the control–alcohol use relationship remains after accounting for levels of secrecy. Point 2 can be ruled out, because the association between drinking and control occurs at all ages. Points 3 and 4 attest to early identification of drinking. Alcohol initiation may lead to a child‐directed process; alternatively, knowledge of a child's drinking may indicate independence and maturity or highlight the futility of the parent's efforts to control behaviour 37. While parents may wish to facilitate adolescent self‐determination, unsupported adolescent decision‐making could actually worsen wellbeing 38; this issue is particularly salient, given social pressures to drink 39, 40, and given that this relationship is present from age 11. Teenagers in 2015 are entering a recommodified alcohol market 41, comparable in social environment to that of their parents, but markedly different in drinking intensity. Parents may endorse drinking autonomy at the same age as was afforded to them, but in so doing are facilitating entry into a markedly different risk environment. Differentially supporting autonomy for identity development in some social arenas, while maintaining control and parental authority in relation to risk issues such as drinking, may represent ‘optimal' support for adolescent development 42.

Parental solicitation showed little association with alcohol outcomes 43. It may be easier to encourage parental behaviour change in terms of initiating conversation than it is to change behaviour surrounding curfews or setting boundaries on adolescent behaviour and whereabouts 44. Parental attachment had little direct influence on alcohol use or parental behaviour; any cross‐sectional associations are not borne out in longitudinal data 28, suggesting that this is a non‐causal association. However, attachment interacts with the reverse causal process whereby alcohol use affects the home environment. A recent model from family systems theory outlined how sibling and parent relationships can lead to substance use problems 45. Understanding a reverse causal process, and how this process differs according to characteristics of the family system, could have implications for integrating external social influences into family therapeutic work.

Considering the developmental and social differences with age, there was surprisingly little evidence for differences in younger or older adolescence. There is a general shift in the importance of family and peers from childhood to adolescence 46, but our results suggest that the role of parents in determining alcohol behaviour is consistently important. A study of parents uncovered concerns about the school and peer context—rather than the family home—as the best place to learn responsible drinking 47; this suggests that parents are receptive to implementing a preventive approach to health risk behaviours. As such, in addition to school, family is an appropriate context in which to deliver preventive interventions 48, 49.

A major strength of the study is its large sample size, providing sufficient power to test for group differences and changes over time. None the less, this study has several limitations. Data were collected from children, so we do not know if parents knew, suspected or permitted their children to drink. Furthermore, school environment may influence alcohol use, and this study provides no insight into the interplay between home and school contexts. Future research could assess in greater detail how characteristics of school interact with family processes to influence drinking behaviour.

Variation in parenting practices may amplify inequalities in life‐style‐related illnesses or social harms in later life, particularly given the socio‐economic patterning in parenting approaches 50. The family environment is thus a potential arena for preventive interventions and policy, but not simply from the traditional social service model of intervening with ‘at‐risk' families 51. The reverse causal processes uncovered in this study highlight the fact that parenting behaviour relates to dynamic family processes, rather than fixed parenting skills and knowledge. Preventive interventions which conceptualize parenting as a modifiable, resource‐based practice 52 may have benefits for family functioning and alcohol outcomes. Given the prevalence of alcohol use in adolescence and among the wider population, broader, resource‐based approaches may realize greater benefits than targeted interventions 53.

Research often endorses the early years as the key target for successful health 54 and social wellbeing 55 policies, advice that has been picked up at the state level, certainly in the United Kingdom 56; family interventions for adolescents have received somewhat less attention. Given that adolescence is the critical period for the beginning of alcohol use, and that the harms of excessive drinking are not confined to children from ‘problem' families, support for adolescent parenting—rather than alcohol awareness for parents—may be a fruitful target for public policy relating to young people's health risk behaviour.

### Declaration of interests

None.

## Supporting information


**Table A1** Number of respondents and cohort characteristics across five years
**Table A2** Time‐varying characteristics across five years
**Table A3** Frequency of alcohol use across five years by gender
**Table A4** Mean (sd.) for parental monitoring subscales by year and gender
**Table A5** Fit statistics for Structural Equation Models using linear alcohol measures
**Table A6** Cross lagged coefficients for the association between alcohol use frequency and parental control: linear alcohol use measure
**Table A7** Cross lagged coefficients for the association between alcohol use frequency and child disclosure: linear alcohol use measure
**Table A8** Cross lagged coefficients for the association between alcohol use frequency and child secrecy: linear alcohol use measure
**Table A9** Cross lagged coefficients for the association between alcohol use frequency and parental solicitation: linear alcohol use measure
**Table A10** Cross lagged coefficients for the association between alcohol use frequency, parental control, child disclosure and child secrecy: linear alcohol use measure
**Table A11** Tests of difference in estimated coefficients over time
**Table A12** Difference between male and female model coefficients, tested jointly and within single years
**Table A13** Difference between coefficients comparing high attachment and low attachment groups, tested jointly and within single years

Supporting info item
