# Supplementary material for: Assessing elements of a family approach to reduce adolescent drinking frequency: parent–adolescent relationship, knowledge management and keeping secrets
Source: Addiction. 2016 Feb 10;111(5):843–53. doi: 10.1111/add.13258 (PMC4949705; doi:10.1111/add.13258)
Supplement: Supplementary file 1 — Supporting info item [file ADD-111-843-s001.docx]

Methods

*The Belfast Youth Development Study*

In year two, several new schools joined that had not been surveyed in year one. Teachers at some schools were participating in industrial action during year four and hence pupils at these schools were not surveyed. In both cases, the reason for missing information can be assumed to be random (MCAR), so multiple imputation can appropriately account for missing data in year one and year four for respondents in these schools.

*Family affluence*

Respondents were asked questions about the number of cars at their household (None, one, two or more), number of family holidays (none, one, two, three or more), parental employment status (None, part-time, full-time for mother and father), whether they had a bedroom to themselves (yes/no), the type of house they lived in (apartment, terraced, semi detached, detached), and eligibility for free school meals (signifying parental receipt of benefits; yes/no). Principal components analyses were used to create affluence measures in each year based on a linear combination of these items. Number of family holidays was dropped, as it loaded onto a separate factor, decision to take holidays appears to be largely independent of socioeconomic position. A single component modelled around 35% of the variance in the affluence indicators (Rho; year 1 0.38; year 2 0.39; year 3 0.35; year 4 0.34; year 5 0.37). In all years, the first component had an eigenvalue between 2.03 (year 4) and 2.31 (year 2). Eigenvalues for all other components fell below one. Affluence factor scores were computed within each year, and to normalise the negative skew, squared scores were used in analysis. Principal components analyses were conducted using Stata 13.1.

*Parental monitoring*

The parental solicitation component included items such as ‘how often do your parents talk with the parents of your friends’, and ‘how often do your parents start a conversation about things that happened during a normal day at school?’.

The parental control component had items including ‘do you need to have your parents’ permission to stay out late on a weekday evening’, and ‘if you have been out late one night do your parents require you to explain what you did and who you were with?’

The child disclosure subscale included questions such as ‘do you talk at home about how you are doing in different subjects at school’

The secrecy subscale included questions such as ‘do you keep a lot of secrets from your parents about what you do in your free time?’

*Statistical analysis*

Multiple imputation models were used to account for missing information on cohort members. In addition to imputing values for alcohol use, monitoring, SDQ, IPPA and affluence scales, the accuracy of the imputation models were enhanced by including: living arrangements, free meal eligibility, ever been drunk (yes/no), and overall monitoring, and the fully observed school level variables, school gender, school town and school management (catholic or state maintained). The Stata MI command suite and Realcom Impute were used to estimate single level and two level (pupils within school) imputation models. As the interpretation of the single level and two level models were comparable, the single level models are presented. The cohort was split into four groups based on gender and high/low attachment before estimating the imputation models, and 50 datasets were created.

To account for those who did not have an observed IPPA score at all three time points, the change in question scaling over time, and to avoid using a ‘median split’ method which would not account for natural changes in parent-child relationships over time, high and low parental attachment groups were determined using a 2 class latent profile model with full information maximum likelihood estimation, modelling responses to IPPA scores in each year. The Entropy (0.65) fit statistic suggested moderate to poor model fit. Adding gender, alcohol use, monitoring, affluence, and SDQ measures increased entropy to 0.77 with little change in the mean ippa scores within each class. As including covariates introduced a greater proportion of missing data, and thus gave predicted class membership for less of the cohort, class membership determined from the unadjusted models was used for analyses.

*Testing gender- and high/low attachment differences*

As Stata does not support multiple group testing for ordinal logistic regression SEM models, continuous versions of the alcohol use frequency variables, scaled between zero and one were calculated as fractional relative rankings [alcohol frequency rank / number of respondents in a given year] for each of the five years (for example, a non-drinker in year 5 was joint 124.5^th^ rank in that year on the ordinal alcohol measure. This rank, divided by 3,491 respondents in year 5 gives a fractional relative ranking of 0.04. The same method gave a ranking of 0.76 for a frequent drinker in the same year).

These were used to test for differences by gender and parental attachment groups. Ordinal regression and linear regression models gave comparable model fit and strength of association for individual parameters, suggesting the use of fractional relative ranks in the models was appropriate.

*Results*

Tables A1 to A4 provide a description of the cohort, and show how the proportion of frequent drinkers changes across the five years of the study. Around 5% of children living with two biological parents drank weekly or more frequently in year one, compared to 7% of children in single parent families, and 8% of those in step-parent families. The relative differential narrowed in later years (year five; biological parent 43%, single parent 58%, step parent 58%. Based on year 1 SDQ cut off scores, those in poorer mental health (18%) were more likely to drink frequently in year two than those in good mental health (13%). A similar pattern appeared relating year four SDQ scores to year five alcohol use (55% and 45%). Before controlling for confounding, children reporting better levels of parental attachment tended not to drink as frequently. Comparing the least securely attached quintile in year one to the most securely attached, the proportions drinking frequently in year two were 19% and 8% respectively. Comparing year the least securely attached quintile in year four to the most securely attached, the proportions of frequent drinkers in year five were 57% and 33% respectively.

Table A1: number of respondents and cohort characteristics across five years

|  | **Total** |
| --- | --- |
| **Total respondents** | 4,937 |
| **Gender** |  |
| Male | 2,348 (47.6) |
| Female | 2,589 (52.4) |
| **Living arrangements** |  |
| Biological parents | 3,545 (71.8%) |
| Step / foster parents | 942 (19.1%) |
| Single parent | 450  (9.1%) |

Table A2: Time-varying characteristics across five years

|  | **Total** | **Year 1** | **Year 2** | **Year 3** | **Year 4** | **Year 5** |
| --- | --- | --- | --- | --- | --- | --- |
| **Total respondents** | 4,937 | 3,681 | 4,101 | 4,334 | 3,797 | 3,607 |
| **Mental Health** |  |  |  |  |  |  |
| SDQ |  |  |  |  |  |  |
| Mean (sd) |  | 11.9 (5.40) |  |  | 11.9 (5.10) |  |
| Normal |  | 3,318 (90.8%) |  |  | 3,484  (91.9%) |  |
| Abnormal |  | 336  (9.2%) |  |  | 308  (8.1%) |  |
| **Parental attachment** Mean (sd.) |  | 29.9 (3.77) |  | 103.4 (21.80) | 102.9 (22.60) |  |
| **Family Affluence** |  |  |  |  |  |  |
| Mean (sd) |  | 27.4 (13.27) | 27.6 (13.72) | 27.4 (12.65) | 27.4 (12.20) | 27.7 (13.30) |
| Free school meals |  |  |  |  |  |  |
| No |  | 2,426  (73%) | 2,870  (74.0%) | 3,057  (76.1%) | 2,784  (79.5%) | 2,730  (80.8%) |
| Yes |  | 891  (27%) | 1,009  (26.0%) | 962  (23.9%) | 719  (20.5%) | 648  (19.2%) |

Table A3: Frequency of alcohol use across five years by gender

| Drinking Frequency | **Males** | **Females** | **Total** |
| --- | --- | --- | --- |
| Year 1 |  |  |  |
| None | 493 (22) | 547 (22) | 1,040 (22) |
| Rarely | 1,103 (49) | 874 (35) | 1,977 (41) |
| Monthly | 92 (4) | 63 (3) | 155 (3) |
| Weekly or more | 135 (6) | 45 (2) | 180 (4) |
| Missing | 434 (19) | 989 (39) | 1,423 (30) |
| **Year 2** | | | |
| None | 607 (27) | 764 (30) | 1,371 (29) |
| Rarely | 749 (33) | 809 (32) | 1,558 (33) |
| Monthly | 224 (10) | 265 (11) | 489 (10) |
| Weekly or more | 270 (12) | 267 (11) | 537 (11) |
| Missing | 407 (18) | 413 (16) | 820 (17) |
| **Year 3** | | | |
| None | 505 (22) | 513 (20) | 1,018 (21) |
| Rarely | 707 (31) | 863 (34) | 1,570 (33) |
| Monthly | 352 (16) | 392 (16) | 744 (16) |
| Weekly or more | 471 (21) | 521 (21) | 992 (21) |
| Missing | 222 (10) | 229 (9) | 451 (9) |
| **Year 4** | | | |
| None | 155 (8) | 147 (6) | 322 (7) |
| Rarely | 545 (24) | 606 (24) | 1,151 (24) |
| Monthly | 362 (16) | 404 (16) | 766 (16) |
| Weekly or more | 552 (24) | 745 (30) | 1,297 (27) |
| Missing | 623 (28) | 616 (24) | 1,239 (26) |
| **Year 5** | | | |
| None | 121 (5) | 120 (5) | 241 (5) |
| Rarely | 370 (16) | 415 (16) | 785 (16) |
| Monthly | 365 (16) | 419 (16) | 784 (16) |
| Weekly or more | 736 (33) | 875 (35) | 1,611 (34) |
| Missing | 665 (29) | 689 (27) | 1,354 (28) |
| **Total** | 2,257 | 2,518 | 4,775 |

Table A4: Mean (sd.) for parental monitoring subscales by year and gender

|  |  | **Year 1** | **Year 2** | **Year 3** | **Year 4** | **Year 5** |
| --- | --- | --- | --- | --- | --- | --- |
| **Females** |  |  |  |  |  |  |
| Solicitation |  | 3.02 (0.93) | 11.63 (4.41) | 11.81 (4.33) | 11.92 (4.43) | 11.74 (4.26) |
| Control |  | 3.75 (1.41) | 13.39 (5.17) | 13.25 (4.97) | 12.96 (5.1) | 12.63 (5.1) |
| Disclosure |  | 2.35 (0.9) | 7.38 (3.34) | 7.01 (3.28) | 6.8 (3.31) | 7.15 (3.18) |
| Secrecy |  | 1.55 (0.74) | 5.19 (2.64) | 4.88 (2.63) | 4.73 (2.64) | 4.67 (2.22) |
| **Males** |  |  |  |  |  |  |
| Solicitation |  | 2.79 (1) | 10.48 (4.61) | 10.16 (4.29) | 10.16 (4.34) | 10.06 (4.21) |
| Control |  | 3.15 (1.56) | 12.03 (5.42) | 11.8 (5.23) | 11.07 (5.28) | 10.58 (5.18) |
| Disclosure |  | 2.12 (0.98) | 6.68 (3.52) | 6.14 (3.27) | 6.01 (3.21) | 6.25 (2.88) |
| Secrecy |  | 1.37 (0.79) | 5.1 (2.53) | 4.76 (2.49) | 4.74 (2.48) | 4.29 (2.01) |
| **Total** |  |  |  |  |  |  |
| Solicitation |  | 2.89 (0.98) | 11.1 (4.54) | 11.03 (4.39) | 11.09 (4.48) | 10.95 (4.32) |
| Control |  | 3.42 (1.53) | 12.75 (5.33) | 12.57 (5.15) | 12.07 (5.27) | 11.67 (5.24) |
| Disclosure |  | 2.22 (0.95) | 7.05 (3.44) | 6.6 (3.3) | 6.43 (3.29) | 6.73 (3.07) |
| Secrecy |  | 1.45 (0.77) | 5.15 (2.59) | 4.83 (2.56) | 4.74 (2.57) | 4.5 (2.14) |

Solicitation and control range 0-5 in year 1; 0-20 in years 2 to 5

Disclosure range 0-3 in year 1; 0-12 in years 2 to 5

Secrecy range 0-2 in year 1; 0-8 in years 2 to 5

The fit coefficients for the complete case models outlined in Tables A6 to A10 appear in Table A5. These values indicate that the models were a reasonably good fit to the data. Tables A6 to A10 below show the results of analysis based on structural equation models using a linear alcohol measure based on fractional relative ranks. Multiple imputation models for fractional relative rank alcohol use (using truncated linear regression), and ordinal alcohol use frequency (ordinal logistic regression) were estimated separately.

Table A5: Fit statistics for Structural Equation Models using linear alcohol measures

| Model | Standardised Root Mean Square Residual | Coefficient of determination |
| --- | --- | --- |
| Table 1 | 0.056 | 0.506 |
| Table 2 | 0.067 | 0.577 |
| Table 3 | 0.065 | 0.541 |
| Table 4 | 0.058 | 0.521 |
| Table 5 | 0.090 | 0.760 |

Table A6: Cross lagged coefficients for the association between alcohol use frequency and parental control: linear alcohol use measure

| Path | Coefficient  (95% CI) | Path | Coefficient  (95% CI) | Path | Coefficient  (95% CI) | Path | Coefficient  (95% CI) |
| --- | --- | --- | --- | --- | --- | --- | --- |
|  |  |  |  |  |  |  |  |
| **Year 2 Alcohol** |  | **Year 3 Alcohol** |  | **Year 4**  **Alcohol** |  | **Year 5 Alcohol** |  |
| Year 1 Control | **-0.032**  **(-0.042, -0.023)** | Year 2 Control | **-0.031**  **(-0.039, -0.024)** | Year 3  Control | **-0.027**  **(-0.035, -0.020)** | Year 4 Control | **-0.019**  **(-0.027, -0.011)** |
|  |  |  |  |  |  |  |  |
| Year 1  Attachment | -0.007  (-0.015, 0.002) | Year 1 Attachment | **-0.014**  **(-0.024, -0.005)** | Year 3  Attachment | -0.004  (-0.014, 0.005) | Year 4 Attachment | -0.004  (-0.014, 0.007) |
|  |  |  |  |  |  |  |  |
| **Year 2 Control** |  | **Year 3 Control** |  | **Year 4**  **Control** |  | **Year 5 Control** |  |
| Year 1 Alcohol | **-0.212**  **(-0.341, -0.084)** | Year 2 Alcohol | **-0.316**  **(-0.429, -0.203)** | Year 3  Alcohol | **-0.215**  **(-0.315, -0.115)** | Year 4 Alcohol | **-0.258**  **(-0.386, -0.13)** |
|  |  |  |  |  |  |  |  |
| Year 1 Attachment | **0.082**  **(0.045, 0.118)** | Year 1 Attachment | **0.096**  **(0.064, 0.128)** | Year 3  Attachment | **0.045**  **(0.01, 0.08)** | Year 4 Attachment | 0.007  (-0.031, 0.044) |

Coefficients adjusted for sex, living arrangements, affluence and mental health

Table A7: Cross lagged coefficients for the association between alcohol use frequency and child disclosure: linear alcohol use measure

| Path | Coefficient  (95% CI) | Path | Coefficient  (95% CI) | Path | Coefficient  (95% CI) | Path | Coefficient  (95% CI) |
| --- | --- | --- | --- | --- | --- | --- | --- |
|  |  |  |  |  |  |  |  |
| **Year 2 Alcohol** |  | **Year 3 Alcohol** |  | **Year 4**  **Alcohol** |  | **Year 5 Alcohol** |  |
| Year 1  Disclosure | -0.033  (-0.044, -0.023) | Year 2  Disclosure | -0.027  (-0.037, -0.017) | Year 3  Disclosure | -0.029  (-0.039, -0.02) | Year 4 Disclosure | -0.013  (-0.023, -0.003) |
| Year 1  Attachment | -0.002  (-0.012, 0.007) | Year 1 Attachment | -0.013  (-0.022, -0.004) | Year 3  Attachment | 0.003  (-0.008, 0.015) | Year 4 Attachment | -0.001  (-0.013, 0.01) |
|  |  |  |  |  |  |  |  |
| **Year 2 Disclosure** |  | **Year 3 Disclosure** |  | **Year 4**  **Disclosure** |  | **Year 5 Disclosure** |  |
| Year 1 Alcohol | -0.317  (-0.466, -0.168) | Year 2 Alcohol | -0.251  (-0.353, -0.148) | Year 3  Alcohol | -0.221  (-0.336, -0.107) | Year 4 Alcohol | -0.251  (-0.359, -0.143) |
| Year 1 Attachment | 0.131  (0.095, 0.167) | Year 1 Attachment | 0.132  (0.096, 0.168) | Year 3  Attachment | 0.12  (0.084, 0.156) | Year 4 Attachment | 0.189  (0.153, 0.225) |

Coefficients adjusted for sex, living arrangements, affluence and mental health

Table A8: Cross lagged coefficients for the association between alcohol use frequency and child secrecy: linear alcohol use measure

| Path | Coefficient  (95% CI) | Path | Coefficient  (95% CI) | Path | Coefficient  (95% CI) | Path | Coefficient  (95% CI) |
| --- | --- | --- | --- | --- | --- | --- | --- |
|  |  |  |  |  |  |  |  |
| **Year 2 Alcohol** |  | **Year 3 Alcohol** |  | **Year 4 Alcohol** |  | **Year 5 Alcohol** |  |
| Year 1  Secrecy | **-0.047**  **(-0.057, -0.037)** | Year 2  Secrecy | **-0.029**  **(-0.038, -0.02)** | Year 3  Secrecy | **-0.034**  **(-0.043, -0.025)** | Year 4 Secrecy | **-0.018**  **(-0.028, -0.008)** |
| Year 1  Attachment | -0.001  (-0.01, 0.008) | Year 1 Attachment | **-0.015**  **(-0.024, -0.006)** | Year 3 Attachment | 0.00  (-0.01, 0.009) | Year 4 Attachment | -0.001  (-0.012, 0.010) |
|  |  |  |  |  |  |  |  |
| **Year 2 Secrecy** |  | **Year 3 Secrecy** |  | **Year 4 Secrecy** |  | **Year 5 Secrecy** |  |
| Year 1 Alcohol | **-0.475**  **(-0.61, -0.341)** | Year 2 Alcohol | **-0.542**  **(-0.662, -0.422)** | Year 3 Alcohol | **-0.508**  **(-0.632, -0.383)** | Year 4 Alcohol | **-0.525**  **(-0.660, -0.391)** |
| Year 1 Attachment | **0.096**  **(0.058, 0.134)** | Year 1 Attachment | **0.1**  **(0.065, 0.135)** | Year 3 Attachment | **0.140**  **(0.100, 0.179)** | Year 4 Attachment | **0.172**  **(0.132, 0.212)** |

Higher Secrecy scores indicate greater Disclosure /lower levels of secret-keeping

Coefficients adjusted for sex, living arrangements, affluence and mental health

Table A9: Cross lagged coefficients for the association between alcohol use frequency and parental solicitation: linear alcohol use measure

| Path | Coefficient  (95% CI) | Path | Coefficient  (95% CI) | Path | Coefficient  (95% CI) | Path | Coefficient  (95% CI) |
| --- | --- | --- | --- | --- | --- | --- | --- |
|  |  |  |  |  |  |  |  |
| **Year 2 Alcohol** |  | **Year 3**  **Alcohol** |  | **Year 4**  **Alcohol** |  | **Year 5 Alcohol** |  |
| Year 1 Solicitation | 0.001  (-0.008, 0.010) | Year 2  Solicitation | **-0.010**  **(-0.017, -0.003)** | Year 3  Solicitation | 0.007  (-0.001, 0.016) | Year 4 Solicitation | 0.000  (-0.010, 0.009) |
| Year 1  Attachment | **-0.014**  **(-0.023, -0.004)** | Year 1  Attachment | **-0.017**  **(-0.027, -0.008)** | Year 3  Attachment | **-0.013**  **(-0.024, -0.003)** | Year 4 Attachment | -0.007  (-0.018, 0.004) |
|  |  |  |  |  |  |  |  |
| **Year 2 Solicitation** |  | **Year 3**  **Solicitation** |  | **Year 4**  **Solicitation** |  | **Year 5 Solicitation** |  |
| Year 1 Alcohol | **-0.219**  **(-0.369, -0.068)** | Year 2  Alcohol | -0.029  (-0.142, 0.084) | Year 3  Alcohol | -0.007  (-0.125, 0.111) | Year 4 Alcohol | 0.0948  (-0.0189,0.2085) |
| Year 1 Attachment | **0.131**  **(0.089, 0.173)** | Year 1  Attachment | **0.132**  **(0.095, 0.168)** | Year 3  Attachment | **0.114**  **(0.077, 0.152)** | Year 4 Attachment | **0.0707**  **(0.0321,0.1093)** |

Coefficients adjusted for sex, living arrangements, affluence and mental health

Table A10: Cross lagged coefficients for the association between alcohol use frequency, parental control, child disclosure and child secrecy: linear alcohol use measure

| **Path** | **Coefficient**  **(95% CI)** | **Path** | **Coefficient**  **(95% CI)** | **Path** | **Coefficient**  **(95% CI)** | **Path** | **Coefficient**  **(95% CI)** |
| --- | --- | --- | --- | --- | --- | --- | --- |
|  |  |  |  |  |  |  |  |
| **Year 2 Alcohol** |  | **Year 3**  **Alcohol** |  | **Year 4**  **Alcohol** |  | **Year 5 Alcohol** |  |
| Year 1  Control | **-0.022**  **(-0.032, -0.012)** | Year 2 Control | **-0.024**  **(-0.032, -0.016)** | Year 3  Control | **-0.02**  **(-0.028, -0.011)** | Year 4 Control | **-0.017**  **(-0.026, -0.008)** |
| Year 1 Disclosure | **-0.014**  **(-0.025, -0.002)** | Year 2 Disclosure | **-0.011**  **(-0.021, 0.00)** | Year 3 Disclosure | **-0.015**  **(-0.026, -0.004)** | Year 4 Disclosure | -0.003  (-0.013, 0.008) |
| Year 1  Secrecy | **-0.038**  **(-0.048, -0.028)** | Year 2  Secrecy | **-0.022**  **(-0.032, -0.013)** | Year 3  Secrecy | **-0.029**  **(-0.038, -0.02)** | Year 4 Secrecy | **-0.016**  **(-0.027, -0.006)** |
| Year 1  Attachment | 0.006  (-0.004, 0.015) | Year 1  Attachment | **-0.01**  **(-0.019, 0.00)** | Year 3  Attachment | 0.01  (-0.001, 0.021) | Year 4 Attachment | 0.003  (-0.009, 0.014) |
|  |  |  |  |  |  |  |  |
| **Year 2 Control** |  | **Year 3**  **Control** |  | **Year 4 Control** |  | **Year 5 Control** |  |
| Year 1 Alcohol | **-0.133**  **(-0.261, -0.004)** | Year 2  Alcohol | **-0.207**  **(-0.326, -0.089)** | Year 3  Alcohol | **-0.125**  **(-0.23, -0.02)** | Year 4 Alcohol | **-0.202**  **(-0.340, -0.064)** |
| Year 1 Disclosure | **0.058**  **(0.013, 0.102)** | Year 2 Disclosure | **0.066**  **(0.034, 0.098)** | Year 3 Disclosure | **0.087**  **(0.046, 0.128)** | Year 4 Disclosure | **0.559**  **(0.517, 0.600)** |
| Year 1  Secrecy | **0.065**  **(0.025, 0.105)** | Year 2  Secrecy | **0.052**  **(0.024, 0.079)** | Year 3  Secrecy | **0.043**  **(0.01, 0.077)** | Year 4 Secrecy | **0.065**  **(0.025, 0.104)** |
| Year 1 Attachment | **0.051**  **(0.011, 0.091)** | Year 1  Attachment | **0.077**  **(0.044, 0.111)** | Year 3  Attachment | -0.002  (-0.042, 0.039) | Year 4 Attachment | -0.028  (-0.07, 0.014) |
|  |  |  |  |  |  |  |  |
| **Year 2 Disclosure** |  | **Year 3 Disclosure** |  | **Year 4**  **Disclosure** |  | **Year 5 Disclosure** |  |
| Year 1 Alcohol | **-0.186**  **(-0.34, -0.033)** | Year 2  Alcohol | **-0.118**  **(-0.233, -0.003)** | Year 3  Alcohol | -0.089  (-0.207, 0.029) | Year 4 Alcohol | -0.085  (-0.204, 0.035) |
| Year 1 Control | **0.108**  **(0.068, 0.148)** | Year 2 Control | **0.114**  **(0.078, 0.151)** | Year 3  Control | **0.095**  **(0.061, 0.129)** | Year 4  Control | 0.017  (-0.014, 0.049) |
| Year 1  Secrecy | **0.108**  **(0.071, 0.145)** | Year 2  Secrecy | **0.085**  **(0.046, 0.124)** | Year 3  Secrecy | **0.08**  **(0.047, 0.112)** | Year 4 Secrecy | **0.153**  **(0.113, 0.193)** |
| Year 1 Attachment | **0.103**  **(0.068, 0.139)** | Year 1  Attachment | **0.117**  **(0.08, 0.155)** | Year 3  Attachment | **0.102**  **(0.064, 0.139)** | Year 4 Attachment | **0.151**  **(0.115, 0.186)** |
|  |  |  |  |  |  |  |  |
| **Year 2 Secrecy** |  | **Year 3 Secrecy** |  | **Year 4 Secrecy** |  | **Year 5 Secrecy** |  |
| Year 1 Alcohol | **-0.412**  **(-0.55, -0.274)** | Year 2  Alcohol | **-0.426**  **(-0.548, -0.305)** | Year 3  Alcohol | **-0.428**  **(-0.556, -0.300)** | Year 4 Alcohol | **-0.378**  **(-0.513, -0.243)** |
| Year 1 Control | **0.059**  **(0.017, 0.100)** | Year 2 Control | 0.034  (-0.002, 0.07) | Year 3  Control | 0.024  (-0.014, 0.062) | Year 4  Control | **0.057**  **(0.022, 0.092)** |
| Year 1 Disclosure | **0.067**  **(0.025, 0.108)** | Year 2 Disclosure | **0.161**  **(0.12, 0.202)** | Year 3 Disclosure | **0.109**  **(0.069, 0.149)** | Year 4 Disclosure | **0.233**  **(0.190, 0.276)** |
| Year 1 Attachment | **0.07**  **(0.033, 0.108)** | Year 1  Attachment | **0.063**  **(0.026, 0.099)** | Year 3  Attachment | **0.09**  **(0.047, 0.132)** | Year 4 Attachment | **0.07**  **(0.029, 0.112)** |

Coefficients adjusted for sex, living arrangements, affluence and mental health

Table A11: Tests of difference in estimated coefficients over time

| **Path** | **Coefficient**  **(95% CI)** | **P value** |
| --- | --- | --- |
|  |  |  |
| **Alcohol** |  |  |
| Control -> Alcohol |  |  |
| All time points |  | 0.29 |
| Year 4 -> 5 - Year 3 -> 4 | 0.00 (-0.02, 0.01) | 0.67 |
| Year 3 -> 4 - Year 2 -> 3 | 0.01 (0.00, 0.02) | 0.14 |
| Year 2 -> 3 - Year 1 -> 2 | 0.0 (-0.02, 0.01) | 0.86 |
| Disclosure -> Alcohol |  |  |
| All time points |  | 0.53 |
| Year 4 -> 5 - Year 3 -> 4 | 0.01 (-0.01, 0.02) | 0.40 |
| Year 3 -> 4 - Year 2 -> 3 | 0.00 (-0.02, 0.02) | 0.98 |
| Year 2 -> 3 - Year 1 -> 2 | 0.00 (-0.01, 0.02) | 0.75 |
| Secrecy -> Alcohol |  |  |
| All time points |  | 0.02 |
| Year 4 -> 5 - Year 3 -> 4 | 0.01 (-0.01, 0.02) | 0.22 |
| Year 3 -> 4 - Year 2 -> 3 | 0.00 (-0.02, 0.01) | 0.31 |
| Year 2 -> 3 - Year 1 -> 2 | 0.02 (0.00, 0.03) | 0.02 |
| **Control** |  |  |
| Alcohol -> Control |  |  |
| All time points |  | 0.18 |
| Year 4 -> 5 - Year 3 -> 4 | 0.11 (-0.06, 0.29) | 0.20 |
| Year 3 -> 4 - Year 2 -> 3 | -0.13 (-0.30, 0.03) | 0.12 |
| Year 2 -> 3 - Year 1 -> 2 | -0.04 (-0.22, 0.14) | 0.65 |
| Disclosure -> Control |  |  |
| All time points |  | 0.96 |
| Year 4 -> 5 - Year 3 -> 4 | 0.02 (-0.04, 0.08) | 0.61 |
| Year 3 -> 4 - Year 2 -> 3 | -0.01 (-0.07, 0.04) | 0.67 |
| Year 2 -> 3 - Year 1 -> 2 | 0.00 (-0.06, 0.05) | 0.93 |
| Secrecy -> Control |  |  |
| All time points |  | 0.09 |
| Year 4 -> 5 - Year 3 -> 4 | 0.03 (-0.02, 0.08) | 0.22 |
| Year 3 -> 4 - Year 2 -> 3 | -0.06 (-0.11, -0.01) | 0.02 |
| Year 2 -> 3 - Year 1 -> 2 | 0.00 (-0.05, 0.04) | 0.86 |
| **Disclosure** |  |  |
| Alcohol -> Disclosure |  |  |
| All time points |  | 0.17 |
| Year 4 -> 5 - Year 3 -> 4 | 0.16 (0.01, 0.32) | 0.04 |
| Year 3 -> 4 - Year 2 -> 3 | -0.11 (-0.29, 0.07) | 0.12 |
| Year 2 -> 3 - Year 1 -> 2 | 0.07 (-0.10, 0.23) | 0.44 |
| Control -> Disclosure |  |  |
| All time points |  | <0.001 |
| Year 4 -> 5 - Year 3 -> 4 | -0.04 (-0.09, 0.01) | 0.09 |
| Year 3 -> 4 - Year 2 -> 3 | -0.03 (-0.08, 0.02) | 0.22 |
| Year 2 -> 3 - Year 1 -> 2 | -0.02 (-0.07, 0.04) | 0.55 |
| Secrecy -> Disclosure |  |  |
| All time points |  | <0.001 |
| Year 4 -> 5 - Year 3 -> 4 | 0.12 (0.06, 0.17) | 0 |
| Year 3 -> 4 - Year 2 -> 3 | -0.05 (-0.10, -0.01) | 0.03 |
| Year 2 -> 3 - Year 1 -> 2 | -0.02 (-0.07, 0.04) | 0.56 |
| **Secrecy** |  |  |
| Alcohol -> Secrecy |  |  |
| All time points |  | 0.33 |
| Year 4 -> 5 - Year 3 -> 4 | 0.16 (-0.01, 0.33) | 0.07 |
| Year 3 -> 4 - Year 2 -> 3 | -0.11 (-0.28, 0.06) | 0.21 |
| Year 2 -> 3 - Year 1 -> 2 | 0.00 (-0.20, 0.20) | 0.99 |
| Control -> Secrecy |  |  |
| All time points |  | 0.14 |
| Year 4 -> 5 - Year 3 -> 4 | 0.05 (0.00, 0.10) | 0.07 |
| Year 3 -> 4 - Year 2 -> 3 | -0.02 (-0.07, 0.04) | 0.58 |
| Year 2 -> 3 - Year 1 -> 2 | -0.03 (-0.09, 0.03) | 0.26 |
| Disclosure -> Secrecy |  |  |
| All time points |  | <0.001 |
| Year 4 -> 5 - Year 3 -> 4 | 0.15 (0.09, 0.20) | 0.00 |
| Year 3 -> 4 - Year 2 -> 3 | -0.07 (-0.12, -0.01) | 0.02 |
| Year 2 -> 3 - Year 1 -> 2 | 0.09 (0.03, 0.14) | 0.00 |

Table A12: Difference between male and female model coefficients, tested jointly and within single years

| **Path** | **Coefficient**  **(95% CI)** | **P value** |
| --- | --- | --- |
|  |  |  |
| **Alcohol** |  |  |
| Control -> Alcohol |  | 0.41 |
| Year 4 -> 5 | 0.00 (-0.01, 0.02) | 0.71 |
| Year 3 -> 4 | 0.02 (0.00, 0.04) | 0.05 |
| Year 2 -> 3 | 0.00 (-0.02, 0.01) | 0.84 |
| Year 1 -> 2 | 0.01 (-0.01, 0.03) | 0.58 |
| Disclosure -> Alcohol |  | 0.22 |
| Year 4 -> 5 | 0.00 (-0.02, 0.02) | 0.83 |
| Year 3 -> 4 | 0.02 (0.00, 0.04) | 0.06 |
| Year 2 -> 3 | 0.00 (-0.02, 0.02) | 0.93 |
| Year 1 -> 2 | -0.02 (-0.04, 0.01) | 0.14 |
| Secrecy -> Alcohol |  | 0.11 |
| Year 4 -> 5 | 0.01 (-0.01, 0.03) | 0.26 |
| Year 3 -> 4 | 0.00 (-0.02, 0.02) | 0.8 |
| Year 2 -> 3 | 0.02 (0.00, 0.04) | 0.05 |
| Year 1 -> 2 | 0.01 (-0.01, 0.04) | 0.15 |
| **Control** |  |  |
| Alcohol -> Control |  | 0.08 |
| Year 4 -> 5 | -0.22 (-0.45, 0.02) | 0.07 |
| Year 3 -> 4 | -0.23 (-0.44, -0.03) | 0.03 |
| Year 2 -> 3 | -0.13 (-0.37, 0.11) | 0.29 |
| Year 1 -> 2 | 0.03 (-0.25, 0.30) | 0.85 |
| **Disclosure** |  |  |
| Alcohol -> Disclosure |  | 0.78 |
| Year 4 -> 5 | 0.03 (-0.17, 0.23) | 0.78 |
| Year 3 -> 4 | -0.12 (-0.36, 0.12) | 0.32 |
| Year 2 -> 3 | -0.08 (-0.33, 0.17) | 0.54 |
| Year 1 -> 2 | 0.06 (-0.24, 0.36) | 0.7 |
| **Secrecy** |  |  |
| Alcohol -> Secrecy |  |  |
| All time points |  | 0.97 |
| Year 4 -> 5 | -0.06 (-0.32, 0.20) | 0.65 |
| Year 3 -> 4 | 0.06 (-0.18, 0.31) | 0.61 |
| Year 2 -> 3 | 0.02 (-0.22, 0.27) | 0.85 |
| Year 1 -> 2 | 0.06 (-0.24, 0.36) | 0.71 |

Table A13: Difference between coefficients comparing high attachment and low attachment groups, tested jointly and within single years

| **Path** | **Coefficient**  **(95% CI)** | **P value** |
| --- | --- | --- |
|  |  |  |
| **Alcohol** |  |  |
| Control -> Alcohol |  |  |
| All time points |  | 0.11 |
| Year 4 -> 5 | -0.01 (-0.03, 0.01) | 0.16 |
| Year 3 -> 4 | 0.01 (0.00, 0.03) | 0.15 |
| Year 2 -> 3 | 0.00 (-0.02, 0.02) | 0.83 |
| Year 1 -> 2 | -0.01 (-0.03, 0.01) | 0.16 |
| Disclosure -> Alcohol |  |  |
| All time points |  | 0.32 |
| Year 4 -> 5 | 0.02 (0.00, 0.04) | 0.05 |
| Year 3 -> 4 | 0.00 (-0.03, 0.02) | 0.71 |
| Year 2 -> 3 | 0.01 (-0.01, 0.03) | 0.33 |
| Year 1 -> 2 | 0.00 (-0.02, 0.03) | 0.8 |
| Secrecy -> Alcohol |  |  |
| All time points |  | 0.60 |
| Year 4 -> 5 | 0.01 (-0.01, 0.03) | 0.39 |
| Year 3 -> 4 | 0.01 (-0.01, 0.03) | 0.26 |
| Year 2 -> 3 | 0.00 (-0.03, 0.02) | 0.65 |
| Year 1 -> 2 | 0.01 (-0.01, 0.03) | 0.56 |
| **Control** |  |  |
| Alcohol -> Control |  |  |
| All time points |  | 0.04 |
| Year 4 -> 5 | -0.35 (-0.61, -0.09) | 0.01 |
| Year 3 -> 4 | 0.13 (-0.12, 0.37) | 0.30 |
| Year 2 -> 3 | 0.02 (-0.26, 0.29) | 0.91 |
| Year 1 -> 2 | 0.06 (-0.22, 0.33) | 0.70 |
| **Disclosure** |  |  |
| Alcohol -> Disclosure |  |  |
| All time points |  | 0.03 |
| Year 4 -> 5 | 0.08 (-0.16, 0.32) | 0.53 |
| Year 3 -> 4 | 0.10 (-0.12, 0.33) | 0.38 |
| Year 2 -> 3 | 0.16 (-0.12, 0.45) | 0.26 |
| Year 1 -> 2 | 0.39 (0.11, 0.68) | 0.01 |
| **Secrecy** |  |  |
| Alcohol -> Secrecy |  |  |
| All time points |  | 0.85 |
| Year 4 -> 5 | 0.05 (-0.20, 0.30) | 0.69 |
| Year 3 -> 4 | -0.09 (-0.35, 0.16) | 0.47 |
| Year 2 -> 3 | -0.09 (-0.35, 0.16) | 0.48 |
| Year 1 -> 2 | -0.09 (-0.38, 0.19) | 0.53 |
